# The impact of sustainable public procurement on corporate ESG performance—The Chinese evidence

**DOI:** 10.1371/journal.pone.0292286

**Published:** 2023-10-19

**Authors:** Runyu Li, Fuguo Cao

**Affiliations:** 1 School of Accountancy, Shandong Youth University of Political Science, Shandong, China; 2 School of Law, Central University of Finance and Economics, Beijing, China; 3 School of Finance and Taxation, Central University of Finance and Economics, Beijing, China; Universiti Kebangsaan Malaysia, MALAYSIA

## Abstract

Public procurement is an important bridge between public demand and market supply and may affect corporate behavior. However, in the advocacy of sustainable development, the extant research has rarely combined sustainable public procurement (SPP) with corporate ESG performance, to explore whether governments have contributed to the development of sustainable corporate performance through their sustainable procurement activities. This paper fills in the gap by matching the actual implementation of SPP of 42,369 projects in China over 2015~2020 with 20,125 corporate ESG performance data, to analyze the relationship between SPP implementation and corporate ESG performance. The results show that the implementation of SPP has a significant positive impact on corporate ESG performance. Further heterogeneity analysis reveals that the impact is stronger in China’s eastern and central regions than in other regions, and corporates at a mature stage are more likely to follow the government sustainable behavior. In addition, the implementation of SPP has a long-term effect on corporate ESG performance. The above findings have important policy implications: firstly, there is a better role for government to play as the “invisible hand”, to participate in the market economy; Specifically, SPP policy should be added to government policy tool box to improve corporate ESG performance in addition to disclosure requirement, and the SPP policy employed should in particular attend to the “missing sectors” of sustainability in SPP for the good of corporate ESG; secondly, the government should implement differentiated policies tailored to the region’s economic development conditions and corporate development characteristics; thirdly, a long-term evaluation mechanism should be established so that the government can play a more long-term demonstration and leading role.

## 1. Introduction

In recent years, countries around the world have increasingly focused on the sustainable development of the economy and society [1–3]. As a major means of public financial support to sustainability, public procurement has become a powerful policy tool [4–6], effectively reflecting national-level policy intentions [7]. This policy intent is evident not only in the utilization of public procurement to enhance financial efficiency but also in leveraging substantial government demand to shape corporate behavior and foster sustainable development. Because of its size and prevalence, public procurement has become a powerful policy tool in China to enhance ecological protection [8], promote corporate innovation [9], foster the development of poverty regions [10], and so on.

Public procurement serves as an important bridge between public demand and market supply, and drives corporate behaviors via its institutional arrangements [11]. In practice, government has already begun to use public procurement to generate improvement of corporate ESG performance. For example, the Procurement Policy Note issued by the UK government in September 2020 (PPN06/20) stated that “suppliers’ social values should be taken into account when awarding central government contracts” [12], to promote the importance and development of ESG through the central public procurement process. However, it can be observed that the role of government in fostering sustainable business has predominantly centered on regulating disclosure requirements to enhance transparency, thereby enabling investors to allocate capital towards sustainable endeavors [13, 14]. The academic debate on the transition to a sustainable economy has also largely ignored the effectiveness of government through its procurement activities, which is an obvious omission [15].

The current paper seeks to fill in the existing gaps in the literature by studying the impact of the government sustainable behavior on corporate sustainable performance from the public procurement perspective. By using the micro database of sustainable public procurement (SPP) implementation at the Chinese provincial level, we combined corporate ESG scores in each province to analyze the impact of public procurement implementation further quantitatively on corporate ESG performance.

The main contributions of this paper are as follows: Firstly, we empirically verify the hypothesis that SPP implementation has substantially positive impact on corporate ESG performance. Secondly, we respond to the call from a prior study [16], and employ Resource Dependency Theory to examine the impact of governments on corporates and their sustainability policies. This paper provides empirical evidence for clarifying the boundary of the government role and examining the guidance of government regulation. Lastly, in terms of research methods, we control the endogeneity by using instrumental variables, Chinese and international ESG Index, corporate CSR data, and changing the model to obtain robust empirical conclusions. As a result, the causal characteristics between the implementation of SPP and the corporate ESG are effectively identified.

The paper is structured as follows: Section 2 reviews the relevant literature. Section 3 presents a theoretical analysis and research hypotheses. Section 4 contains the research data and method. Section 5 includes the results of the article. Finally, section 6 concludes with the implications and limitations of the study, and possible issues for future research.

## 2. Literature review

ESG is the acronym for Environment, Social and Governance, an extension and enrichment of the concept of green and responsible investment, and also an important standard for the international community to measure the level of green and sustainable development of corporates. Some researchers have shown that the ESG concept can motivate stakeholders to focus on long-term value, take on social responsibility, and have a positive social impact [17–19]. Thus, ESG provides a continuous impetus for corporates to fulfill their environment and social responsibility.

Research on the influencing factors of ESG performance has gained considerable popularity recently. ESG is a relatively comprehensive concept that can be influenced by socio-political drivers [20], so it is important to link the concept of ESG to the macroeconomic context, such as the social context and institutional background [21, 22]. Kizys et al. (2021) [23] find that corporates with better ESG performance and more board diversity tended to disclose more information about their ESG performance. The disclosure of ESG information positively impacts corporate operational, financial and market performance significantly [24], reduces the risk of corporate financial irregularities and mitigates information asymmetry [25]. Sun et al. (2019) [26] argue that stronger solvency, better profitability and reasonable capital structure can improve ESG performance. Pozzoli et al. (2022) [27] show a significant positive effect of audit committee independence and expertise on ESG performance. Chen et al. (2022) [28] investigate that the effect of different types of ownership structures on corporate ESG performance. Using cross-country data, DasGupta (2022) [29] finds a strong positive influence of financial performance shortfall on corporate ESG performance.

Additionally, certain researches have also delved into the influencing factors of government-related action on corporate ESG performance. In particular, Baldini et al. (2018) [30] found that, at the national level, political systems (laws and level of corruption), labor systems (labor protection and unemployment), and cultural systems (social cohesion and equal opportunities) significantly affect corporate ESG performance. In addition, government pressure can also affect the quality of corporate ESG-related information disclosure [31]. However, the research of the impact of certain government action, such as public procurement, on ESG performance has been neglected [15]. To be specific, there is a lack of empirical literature examining the impact of SPP on corporate ESG performance, the gap of which this paper seeks to fill in.

## 3. Theory analysis and hypothesis

Resource Dependence Theory (RDT), first introduced by Pfeffer and Salancik in 1978, is a significant framework in organizational theory which primarily focuses on the understanding of the relationship between organizations and their external environment, emphasizing the organization’s dependence on external resources to fulfill its resource needs [32]. RDT suggests that organizations must engage in transactions with other entities that control access to critical resources, and these transactions may include the exchange of money, physical resources, information, expertise or social legitimacy [32]. Although the primary focus of this theory is the interdependence among organizations, we could apply this framework to illustrate the influence of the government action on corporate behavior.

The government is the representative of the state and it controls important resources such as laws, money, taxes, etc. According to RDT, government as buyers, controls resources on which suppliers are dependent, hence buying organizations are able to influence the behavior of suppliers [33, 34]. The resources exchange relationship drives the relationship between government and corporates. Corporates need government support, such as fiscal fund, and regulation, such as tax incentives, environmental protection policies. Therefore, government can affect corporates directly or indirectly.

In fact, existing literature has already employed RDT to analyze the general relationship between government and corporates. Bacharach and Lawler (1980) [35] combined RDT and Organizational Politics to explore the power relations and resource competition between government and businesses. Oliver (1991) [36] examined the impact of government policies on businesses and explored how companies employ RDT strategies to adapt to evolving institutional environments. Hillman and Wan (2005) [37] utilized RDT to elucidate the degree of business dependence on the government in different countries and the government influence on their operations.

In recent years, RDT has been adopted to study the organizational interaction in sustainability in particular. Xu et al. (2019) [38] uses RDT to investigate various aspects of supply chain integration, innovation, and sustainability policies. In a meta-analysis of RDT studies, it was found that RDT can explain organizational actions that have societal acceptance rather than economic performance as an ulterior motive [39]. Wontner et al. (2020) [16] used RDT to analyze the extent of influence of resource dependence on the implementation of Community Benefits (CBs) procurement.

Given the above, it is submitted that RDT can be employed to examine the impact of governments on corporates and their sustainability policies, in particular, the impact of SPP on corporate ESG performance. Towards this end, we divided corporates into two groups: those involved in public procurement orders and those not. For those involved in public procurement orders, government sustainable procurement behavior may directly affect corporate behavior in two ways. Firstly, as the largest single consumer in China, public procurement can guide the production of corporates [40], and increase the demand for green products or services [41]. This gives public procurement an unparalleled advantage in guiding corporate production, consumption, and behavior [42]. Secondly, public procurement can regulate corporate behavior by enforcing mandatory requirements. Mandatory regulations for public procurement often encompass employment support for disabled, ethnic minorities and disadvantaged groups [43, 44]; they also include conditions such as occupational safety, payment of social security and good records in credit systems, thus contributing to the promotion of corporate social responsibility [45].

For the corporates that do not participate in public procurement orders, SPP can also indirectly affect their behavior through policy guidance. Some policy guidance in public procurement has the function of market choice, especially in strengthening ecological protection [46], promoting innovation [47–49], improving technological capability [50], etc.

**Fig 1:**
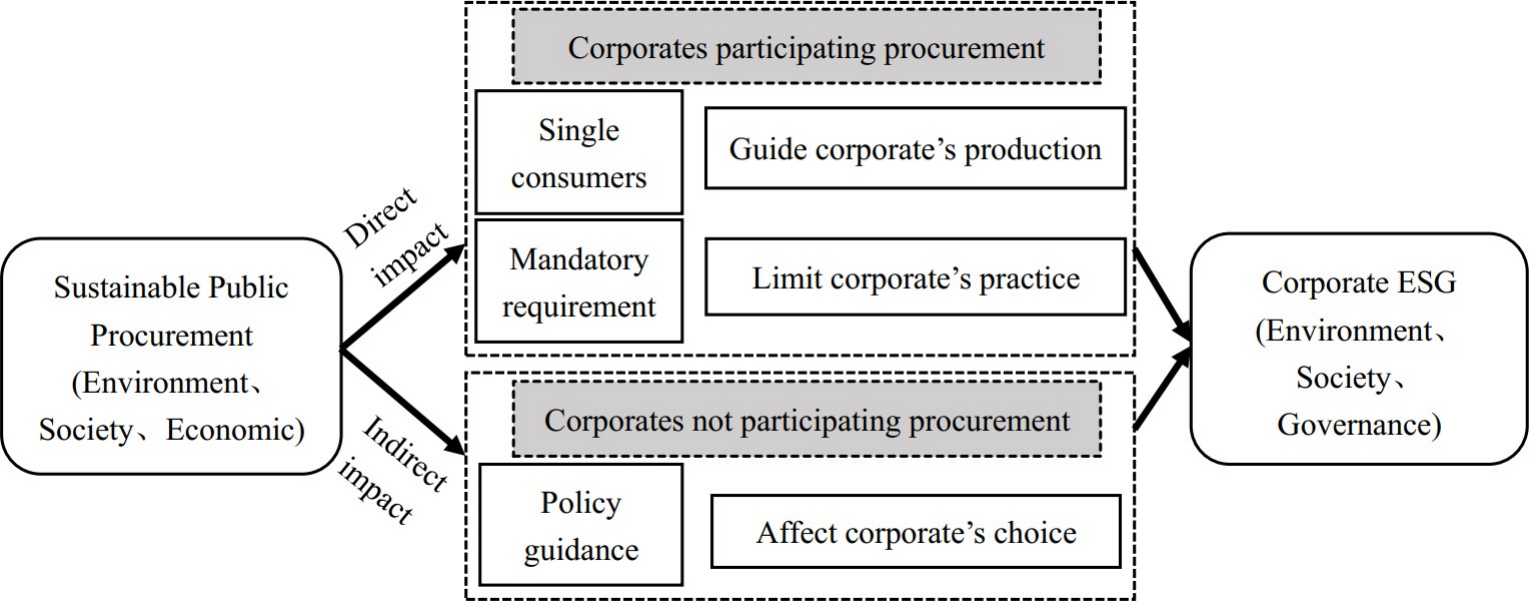
The effect of SPP on corporate ESG performance.

Based on the above analysis, it can be submitted that SPP will affect the sustainability-related performance of corporates whether the corporates participate in the procurement or not (Fig 1). Therefore, this paper further empirically tests the hypothesis:

Implementing SPP will positively affect the corporate ESG performance.

## 4. Data and method

### 4.1 Research model

To explore the relationship between SPP and corporate ESG performance, we develop a basic estimation model as follows:

$${ESG}_{it}=\alpha +\beta {SPP}_{it}+\gamma {Control}_{it}+\Sigma Year+\Sigma Ind+\varepsilon_{it}$$ (1)

Among them, *i* represents the corporate, *t* represents the year, the dependent variable *SPP_it_* represents the average implementation of SPP in the province of corporate *i* in year *t*, *ESG_it_* represents the ESG performance of corporate *i* in year *t*. Control variables include factors that will affect the ESG performance at the corporate level. In addition, we add year and industry dummy variables to control the influence of year and industry factors, *ε* denotes constant and residual terms. We focus on whether the coefficient is significant, that is, whether the implementation of SPP has an impact on the ESG performance of corporates. If *β* is significantly positive, it indicates that the implementation of SPP can contribute to the improvement of corporate ESG performance, and if *β* is negative, it indicates a negative effect.

### 4.2 Research data

#### 4.2.1 Dependent variable

The corporate ESG performance data in this paper is based on the results provided by Sino-Securities ESG Index. We chose this ESG Index because of its alignment with local characteristics, evaluation indicators and data scope.

Firstly, the Sino-Securities ESG Index is more suitable for exploring the relationship between SPP and corporate behavior in the Chinese context compared to other ESG data measured by foreign institutions. The Sino-Securities ESG Index considers the realities of ESG investment, ESG practices, ESG disclosure and ESG policies in China, and some of the indicators carry local characteristics [51].

Secondly, in terms of evaluation indicators, the content and classification of the Sino-Securities ESG Index also correspond to the connotation of SPP assessment carried out for the purpose of this study. For example, by reading and collating the specific ESG indicators of other rating results, we found that compared with other ESG performance results, the Sino-Securities ESG Index takes into account factors related to poverty reduction (Table 1), which is consistent with the important secondary category of poverty reduction in SPP assessment [52]. Therefore, the Sino-Securities ESG Index corresponds to the core of this paper’s SPP study.

**Table 1:** ESG indicator content.

3 Elements	14 Themes	26 Key Indicators
Environment	Environmental Management System	Environmental Management System
	Green business goals	A low-carbon plan or goal
		Green business plan
	Green products	Carbon footprint
		A sustainable product or service
	External environment certification	Environmental certification of the product or corporate
	Environmental violations	Environmental violations
Society	Institution System	Quality of social responsibility reports
	Health and safety	A goal or plan to reduce safety incidents
		Negative business events
		Trend of business accidents
	Social contribution	Social responsibility related donations
		Employee growth rate
		Rural revitalization
	Quality Management	The product or the corporate obtains the quality certification
Governance	Institution building	Corporate self-ESG supervision
	Governance structure	Related party transactions
		Board independence
	Business activities	Tax Transparency
	Operational risk	Asset quality
		Overall financial credibility
		Short-term debt servicing risk
		Risk of equity pledge
		Quality of information disclosure
	External sanctions	Listed Corporates and subsidiaries violations of the law
		Violation of rules and regulations by senior management and shareholders

Thirdly, the scope and coverage of data of the Index can be aligned with that of our SPP study [52]. The Sino-Securities ESG Index results can completely include the six years from 2015 to 2020, and the scope of corporates covers all Chinese listed corporates, while the ESG data from some other rating agencies is not suffice to cover the full period, and only include part of listed corporates.

For better use of the Sino-Securities ESG Index, a nine-point scale is used in this study, to assign scores to the different levels of ESG performance, with higher scores representing better ESG performance (Table 2). These rules are similar to those applied in Lin et al. (2021) [53], Li and Li (2022) [54], and Lian et al. (2023) [55].

**Table 2:** ESG indicator ratings.

Scores	Assignment	Level	Range of scores
AAA	9	Lead	[95,100]
AA	8	Lead	[90,95]
A	7	Lead	[85,90]
BBB	6	Average	[80,85]
BB	5	Average	[75,80]
B	4	Average	[70,75]
CCC	3	Behind	[65,70]
CC	2	Behind	[60,65]
C	1	Behind	[0,60]

#### 4.2.2 Independent and control variable

The implementation of SPP in this paper is based on Cao et al. (2022) [52]. Cao et al. (2022) [52] divided Chinese SPP into 7 main categories: environmentally friendly procurement, circular economy, social return on investment, ethical trade, SMEs-oriented procurement, innovation-oriented procurement and sustainable labeling. Moreover, they identified 26 secondary categories and 57 tertiary categories corresponding to the main categories. They used the web crawler technology to obtain all central-level public procurement tender documents published from 2015 to 2020 on the Chinese Public procurement Network (http://www.ccgp.gov.cn/). After data cleaning and collation, 42,369 central procurement documents were used as the study samples. Finally, they used the text-mining method to assess the sustainable related keywords in the documents and the implementation of SPP in China.

In addition to the demonstration effect of SPP, corporate ESG performance may be influenced by other factors. Research shows that strong solvency, profitability and reasonable capital structure can improve the ESG performance of corporates [26]. Nie et al. (2022) [56] found that the main factors that affect ESG scores include corporate age, debt-to-equity ratio, and equity concentration. In addition, corporates with good ESG performance disclose more information, and board diversity also promotes ESG disclosure [23]. Therefore, this paper uses Tobin’s Q (tobinq), corporate net profit to total assets (profit), corporate age (age), the gearing ratio (ass_lia), cash to total assets (cash_ratio) and top shareholder ownership ratio (big_ratio) to control for the effects of these influencing factors on corporate ESG performance respectively.

**Fig 2:**
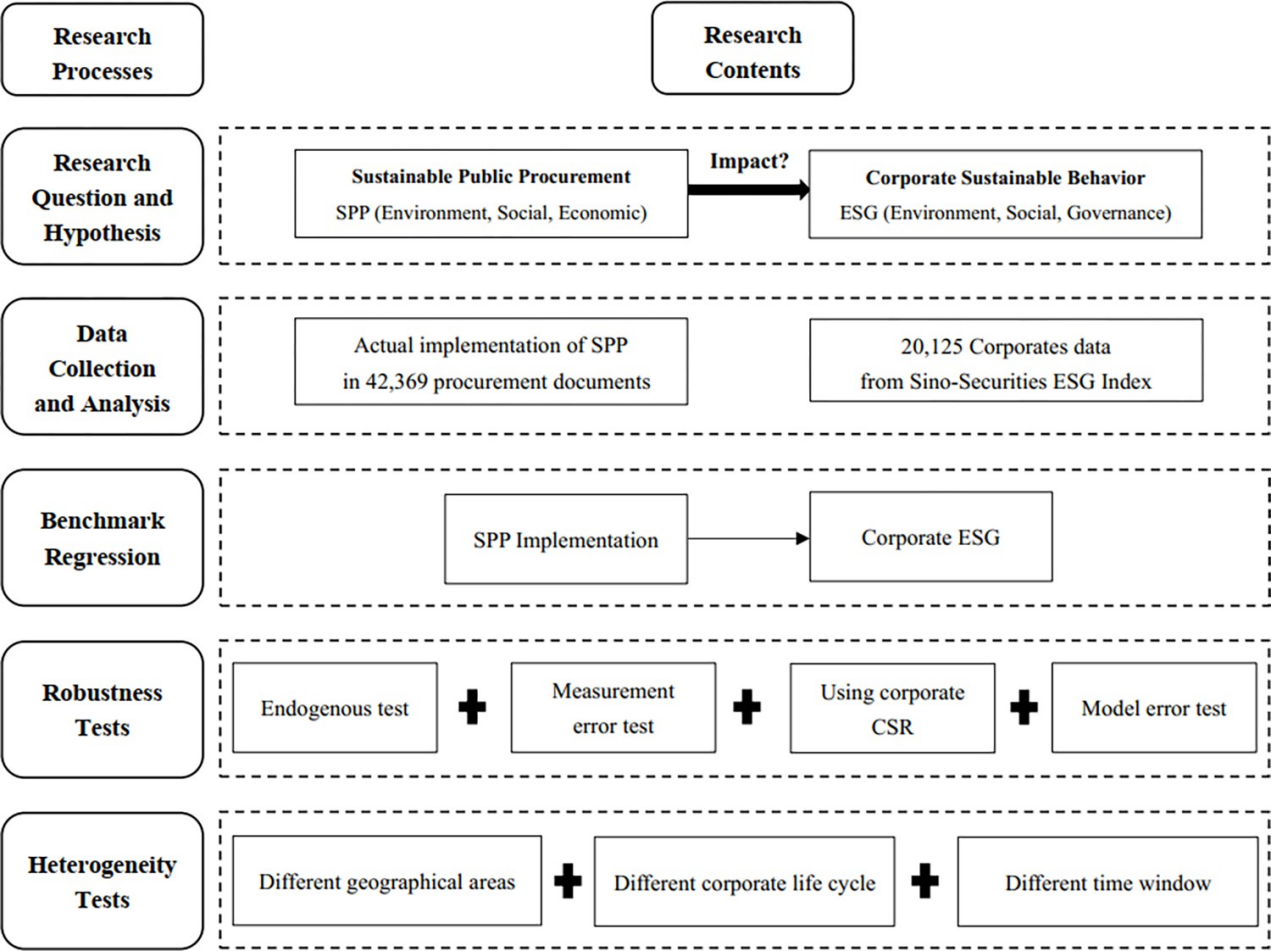
Research strategy.

All other corporate data were obtained from the CSMAR database. Based on the need of the research, the sample was also screened and processed in the following order: (1) excluding ST and *ST categories; (2) excluding samples with asset-to-liability ratios more than 1; (3) excluding samples with missing data. The final sample of 20,125 corporate observations from 31 provinces and autonomous regions was obtained over the six-year period from 2015 to 2020. The specific characteristics of all variables are shown in Table 3. The research strategy for the paper is shown in Fig 2.

**Table 3:** Descriptive statistics.

Variable name	Variable	Obs	Mean	Std. Dev.	Min	Max
SPP	spp	19,858	0.803277	.1910281	0	1
Corporate ESG	esg	20,125	6.41605	1.197614	1	9
Tobin Q	tobinq	17,458	2.236176	2.688601	.673522	122.1895
Net profit as a percentage of its total assets	profit	20,120	.0241686	.3990978	-30.9587	7.446082
Corporate age	age	20,125	11.61118	7.901169	1	31
Asset-liability ratio	ass_lia	17,458	.4251263	.2010282	.008359	.997603
Cash as a percentage of total assets	cash_ratio	20,125	.1536131	.1203403	.000052	.977878
The largest shareholder holding ratio	big_ratio	20,125	33.30148	14.73361	0.29	89.99

## 5. Results

### 5.1 SPP and corporate ESG

This paper uses a regression model to estimate the impact of SPP on corporate ESG performance, and the results are shown in Table 4. Among them, column (1) does not include control variables, does not control for year fixed effects and industry fixed effects, column (2) does not include control variables and includes control effects for year and industry, column (3) includes only year fixed effects, column (4) includes only industry fixed effects, and column (5) controls for both year fixed effects and industry fixed effects, and clusters at the corporate level. The results show that the estimated coefficients of both SPP implementation and corporate ESG performance are highly significant positive (1% significance level). After controlling for year-fixed and industry-fixed effects, the implementation of SPP increases by 1 percentage point, contributing to a 0.18% increase in corporate ESG ratings. The study shows that SPP will promote corporate ESG performance, that is, SPP does have a positive impact on corporate sustainable performance. The hypothesis is thus verified.

**Table 4:** Results of baseline regression.

	(1)	(2)	(3)	(4)	(5)
VARIABLES	esg	esg	esg	esg	esg
spp	0.274***	0.182***	0.193***	0.213***	0.181***
	(0.044)	(0.045)	(0.048)	(0.043)	(0.051)
tobinq			-0.038***	-0.029***	-0.034***
			(0.003)	(0.003)	(0.006)
profit			1.955***	2.031***	2.016***
			(0.092)	(0.091)	(0.228)
age			0.032***	0.023***	0.023***
			(0.001)	(0.001)	(0.003)
ass_lia			0.342***	0.232***	0.219*
			(0.049)	(0.051)	(0.119)
cash_ratio			0.839***	0.525***	0.511***
			(0.081)	(0.082)	(0.146)
big_ratio			0.010***	0.009***	0.008***
			(0.001)	(0.001)	(0.001)
Industry fixed effect	No	Yes	No	Yes	Yes
Year fixed effect	No	Yes	Yes	No	Yes
Clustering	No	No	No	No	Yes
Constant	6.196***	5.704***	5.494***	4.770***	4.967***
	(0.037)	(0.126)	(0.074)	(0.137)	(0.131)
Observations	19,858	19,858	17,230	17,230	17,230
R-squared	0.002	0.120	0.112	0.173	0.180

Note

*, ** and *** are significance levels of 10%, 5% and 1% respectively, with robust standard errors in brackets.

From the coefficient of the controlled variables, we can see that the ratio of assets to liabilities, the ratio of cash to total assets, and the ratio of net profit to total assets have a significant positive impact on ESG performance. Specifically, corporates with lower debt, higher profitability and more cash flow, are more willing and able to respond to the government sustainable procurement behavior by investing in environmental protection, social responsibility and corporate management, thereby improving their ESG performance. In contrast, corporates with weak profitability may have a relatively lower level of technology and management, lack a sound R&D and innovation management system, and thus neglect their social responsibility and contribution to the protection of the environment, and therefore the government actions do not have a substantial impact on these corporates and they neglect their ESG development.

In addition, as the age of the corporate increases, ESG performance is better for corporates with a longer operation period. Shorter-lived corporates, with immature operating models, unstable funding sources and greater risk, have a more urgent need for business expansion and access to capital, thus leaving them with fewer resources and energy to invest in ESG improvement. Longer established corporates are more willing to respond positively to the government call to improve their ESG performance to maintain their reputation and corporate image, thus earning them more credibility resources and gaining more revenue to feed their high-quality development.

However, in the benchmark regression results, we found that the market value of the corporate (Tobin Q) was significantly negative to the coefficient of ESG. Although most studies have shown that there is a mutually reinforcing effect between corporate ESG and corporate value [57, 58], some scholars have also found that the higher the corporate value, the worse the ESG performance [59, 60]. Di Giuli and Kostovetsky (2014) [61] also show that ESG performance is negatively correlated with corporate values, as “any benefit that social responsibility brings to stakeholders comes directly at the expense of corporate value.” Although the results of this paper provide some support for this conclusion, the relationship between corporate market value and ESG performance deserves more in-depth research and exploration.

### 5.2 Robustness tests

This section further validates the relationship between SPP and corporate ESG performance by adding instrumental variables, replacing core explanatory variables, analyzing the relationship between SPP and CSR, and replacing regression models to ensure the reliability of the basic conclusions.

#### 5.2.1 Adding instrumental variables

In the benchmark regression model, although this paper controls for the year and industry factors through the fixed effect, and further controls for factors affecting ESG indicators in terms of basic corporate characteristics, the estimation model may still have endogenous problems. As ESG rating involves three dimensions and is closely related to all stages of corporate development, there may have many other factors that can influence corporate ESG performance and it is not possible to list all the explanatory variables in the model.

Therefore, in order to mitigate the endogeneity problem caused by omitted variables, this paper uses instrumental variables for robustness testing. The ratio of greening coverage, the proportion of private corporates and the unemployment rate are used as the instrumental variables of the key explanatory variables of SPP. On the one hand, the ratio of greening coverage, the proportion of private corporates and the unemployment rate of a region influence the implementation of SPP, which satisfies the correlation hypothesis; on the other hand, these three indicators are not directly related to the ESG of individual corporates and satisfy the exogeneity hypothesis. Furthermore, the regression showed a Cragg-Donald Wald f statistic of 551.308, exceeding the Stockyogo Cutoff and passing the weak instrumental variable test. At the same time, the Kleibergen-Paap R K LM statistic value was 113.998, which passed the unrecognized test. As shown in Table 5, the implementation of SPP and the estimated coefficient of ESG is highly significant and positive, indicating that sustainable procurement behavior can effectively promote the performance of corporate ESG. After controlling for the endogenies of the missing variables in the basic regression, the results are still robust.

**Table 5:** Results after adding instrumental variables.

VARIABLES	esg-iv
spp	0.800***
	(0.268)
tobinq	-0.031***
	(0.006)
profit	1.970***
	(0.220)
age	0.032***
	(0.003)
ass_lia	0.352*
	(0.179)
cash_ratio	0.827***
	(0.162)
big_ratio	0.010***
	(0.002)
Industry fixed effect	Yes
Year fixed effect	Yes
Constant	4.807***
	(0.265)
Observations	17,230
R-squared	0.096

Note

*, ** and *** are significance levels of 10%, 5% and 1% respectively, with robust standard errors in brackets.

#### 5.2.2 Replacing core explanatory variables

In this part, we use Bloomberg ESG rating data to replace the original Sino-Securities ESG data to further test the reliability of the conclusions of this paper. Bloomberg is a global provider of business, financial and public finance information and a representative global rating agency. Therefore, using Bloomberg ESG as a proxy variable can provide a different perspective on the ESG evaluation of the same Chinese listed corporates by global ESG rating agencies; in addition, Bloomberg ESG data comes from corporate social responsibility reports, annual reports, corporate websites and Bloomberg’s surveys. The evaluation system is more scientific and comprehensive, and the data sources are more reliable.

Compared with other ESG evaluation systems abroad, the sample size of Chinese listed corporates covered by the Bloomberg ESG score is larger and the year interval is longer [56]. In Table 6, SPP’s positive effect on ESG remains unchanged. Therefore, SPP behavior has a significant positive effect on ESG, the conclusion is robust.

**Table 6:** Results after replacement with Bloomberg ESG Index.

VARIABLES	Bloomberg esg
spp	0.220*
	(0.127)
tobinq	-0.027**
	(0.014)
profit	2.651***
	(0.307)
age	0.031***
	(0.003)
ass_lia	0.873***
	(0.132)
cash_ratio	0.780***
	(0.218)
big_ratio	0.006***
	(0.001)
Industry fixed effect	Yes
Year fixed effect	Yes
Constant	5.518***
	(0.187)
Observations	2,823
R-squared	0.087

Note

*, ** and *** are significance levels of 10%, 5% and 1% respectively, with robust standard errors in brackets.

#### 5.2.3 The regression analysis of CSR

The concept of Corporate Social Responsibility (CSR) was formally introduced in 1953 [62]. According to Bowen, greater social responsibility should be taken since large corporations have greater social and economic influence and can influence citizens’ lives in many ways. Bowen defines CSR as the policies, plans, and actions that corporates take to meet social goals and values. Corporates should develop in a socially responsible manner, taking into account social and environmental impacts in addition to the production and marketing of their products, and taking appropriate action to manage those impacts.

ESG development is based on CSR, both of which emphasize corporate responsibility and require corporates to take social and environmental impacts into account and take action. At the same time, they all emphasize corporate transparency and accountability to monitor the conduct of corporates throughout the production and consumption chain. However, differences between the two indicators in terms of specific practices have become increasingly apparent over time. First of all, the core concept of CSR is evolving, but it still has a clear ethical and philanthropic imprint. “Doing Good” is at the heart of CSR [63], with the practice of fulfilling public expectations and promoting social and economic well-being outside the interests of individuals and corporations. On the other hand, the core of ESG is “Doing Well and Doing Good” [64], that is, corporates care not only about their own interests but sustainable development as well. In addition, CSR tends to encourage voluntary disclosure, while ESG reporting has evolved from semi-mandatory (disclose or explain) to mandatory disclosure. Under the background of higher requirements for information transparency, CSR ratings with voluntary disclosure can no longer meet the needs of the capital markets, and have objectively contributed to the decline of CSR and the rise of ESG [64]. Although ESG has a broader scope of evaluation than CSR, some scholars still use the two concepts interchangeably [17].

In order to further verify the reliability of the conclusions and provide more perspectives for research, the core explanatory variable ESG is replaced by CSR for further testing. In this paper, we choose the CSR score published by the He Xun CSR report as the core explanatory variable. The system assesses shareholder responsibility, employee responsibility, supplier, customer and consumer responsibility, environmental responsibility and social responsibility, each of them sets up secondary and tertiary indicators respectively to make a comprehensive evaluation of CSR.

Table 7 shows that after controlling for industry and year-fixed effects, the coefficient between the implementation of SPP and corporate CSR is significantly positive. Other control variables in the model did not differ significantly from those in column (5) of the baseline regression in Table 4. Although the focus of ESG and CSR scores is not the same, they both represent the corporate concern for social responsibility and sustainable behavior in addition to profits, and the implementation of SPP also has a positive impact on corporate CSR and ESG performance.

**Table 7:** Results of SPP and corporate CSR.

VARIABLES	csr
spp	3.270***
	(0.471)
tobinq	-0.224***
	(0.035)
profit	26.782***
	(0.988)
age	0.011**
	(0.014)
ass_lia	2.337***
	(0.550)
cash_ratio	9.913***
	(0.890)
big_ratio	0.074***
	(0.007)
Industry fixed effect	Yes
Year fixed effect	Yes
Constant	9.156***
	(1.491)
Observations	17,171
R-squared	0.131

Note

*, ** and *** are significance levels of 10%, 5% and 1% respectively, with robust standard errors in brackets.

#### 5.2.4 Changing regression model

To avoid the effect of model selection bias on the findings, another regression model was used to test the robustness of the results. Given that the corporate ESG performance is a categorical variable from 1 to 9, classified as ordered discrete data, we choose to use the ordered Probit model to estimate the relationship between SPP implementation and corporate ESG performance. As shown in Table 8, after changing the regression model, SPP still has a significant positive effect on corporate ESG performance, and the regression results of the ordered Probit model are generally consistent with the results in Table 4.

**Table 8:** Results of changing the regression model.

	(1)	(2)	(3)	(4)	(5)
VARIABLES	esg	esg	esg	esg	esg
spp	0.231***	0.167***	0.176***	0.172***	0.172***
	(0.039)	(0.042)	(0.045)	(0.045)	(0.049)
tobinq			-0.036***	-0.034***	-0.034***
			(0.003)	(0.005)	(0.006)
profit			1.797***	1.940***	1.940***
			(0.086)	(0.159)	(0.228)
age			0.031***	0.023***	0.023***
			(0.001)	(0.001)	(0.003)
ass_lia			0.332***	0.219***	0.219*
			(0.046)	(0.052)	(0.113)
cash_ratio			0.815***	0.504***	0.504***
			(0.076)	(0.080)	(0.146)
big_ratio			0.009***	0.008***	0.008***
			(0.001)	(0.001)	(0.001)
Industry fixed effect	No	Yes	No	Yes	Yes
Year fixed effect	No	Yes	Yes	No	Yes
Clustering	No	No	No	No	Yes
Observations	19,858	19,858	17,230	17,230	17,230
Pseudo *R2*	0.0006	0.0444	0.0390	0.0672	0.0672

Note

*, ** and *** are significance levels of 10%, 5% and 1% respectively, with robust standard errors in brackets.

### 5.3 Heterogeneity tests

#### 5.3.1 Differences in geographical areas

There are obvious regional differences in China’s economic level and institutional environment. The eastern and central regions have higher economic development and a better institutional environment [65]. A favorable social environment has led to a stronger social concern for corporates to fulfill their social responsibility and improve corporate governance [66], which makes it easier for SPP practices in the east and central regions to play a leading role in promoting the corporate ESG performance.

In addition, the governments in the eastern and central regions have more financial resources to provide incentives, tax breaks and other policy support for socially responsible corporates [67]. As a result, corporates in these regions are more motivated to improve their ESG performance. However, the relative lack of financial resources in China’s western regions also increases the cost of improving the ESG performance of corporates, leading to lower investment in ESG performance by corporations. Column (3) of Table 9 shows that the regression coefficient for corporates in the western region is negative and insignificant. In contrast, the regression coefficients for corporates in the eastern and central regions are both significantly positive. The impact of SPP on ESG performance is greater in the eastern region of China than in the central region (0.302>0.163).

**Table 9:** Results of heterogeneity analysis based on geographical areas.

	(1)	(2)	(3)
	Eastern	Central	Western
VARIABLES	esg	esg	esg
spp	0.302***	0.163**	-0.035
	(0.101)	(0.066)	(0.205)
tobinq	-0.028***	-0.031***	-0.049***
	(0.007)	(0.007)	(0.017)
profit	2.752***	1.969***	1.541***
	(0.490)	(0.224)	(0.332)
age	0.017**	0.026***	0.018**
	(0.007)	(0.003)	(0.007)
ass_lia	0.096	0.267**	0.159
	(0.309)	(0.134)	(0.188)
cash_ratio	0.589*	0.323*	1.365***
	(0.346)	(0.191)	(0.282)
big_ratio	0.005	0.010***	0.003
	(0.004)	(0.002)	(0.003)
Industry fixed effect	Yes	Yes	Yes
Year fixed effect	Yes	Yes	Yes
Constant	5.135***	4.923***	4.939***
	(0.292)	(0.165)	(0.296)
Observations	12,423	2,588	2,219
R-squared	0.208	0.205	0.255

Note

*, ** and *** are significance levels of 10%, 5% and 1% respectively, with robust standard errors in brackets.

#### 5.3.2 Differences in corporate life cycle

According to the life cycle theory, the corporate, as a special organic organism, undergoes different stages, such as start-up, growth, maturity and decline, and each stage faces different challenges and opportunities. Research shows that corporates are in different stages of the life cycle, and the factors affecting the development of corporates are different, bringing about different economic consequences and mechanisms of action [68, 69]. Therefore, this paper draws on Dickinson’s (2011) [70] cash flow portfolio method to divide corporations into different life stages. As listed corporates have already passed the start-up stage [71], the life cycle of the corporates involved in this paper is divided into three stages: growth stage, maturity stage and decline stage, and then grouped regression is conducted.

Table 10 shows that the regression coefficients of the second column are 0.275, which is significant at the level of 5%, indicating that the sustainable behavior of public procurement will lead to the improvement of ESG performance in the mature stage. The coefficients in columns (1) and (3) are 0.121 and 0.116 respectively, indicating that the government will still have a positive but insignificant effect on the ESG performance in the growth and decline phases.

**Table 10:** Results of heterogeneity analysis based on corporate life cycle.

	(1)	(2)	(3)
	Growing	Maturity	Recession
VARIABLES	esg	esg	esg
spp	0.121	0.275**	0.116
	(0.077)	(0.106)	(0.126)
tobinq	-0.020*	-0.041***	-0.028***
	(0.011)	(0.008)	(0.010)
profit	1.969***	1.618***	2.091***
	(0.169)	(0.487)	(0.385)
age	0.000	0.032***	-0.010
	(0.006)	(0.011)	(0.023)
ass_lia	0.016	0.333	0.160
	(0.096)	(0.218)	(0.241)
cash_ratio	0.444***	0.720**	0.040
	(0.126)	(0.334)	(0.263)
big_ratio	0.005***	0.011***	0.010***
	(0.002)	(0.002)	(0.003)
Industry fixed effect	Yes	Yes	Yes
Year fixed effect	Yes	Yes	Yes
Constant	4.677***	4.782***	5.473***
	(0.188)	(0.246)	(0.545)
Observations	9,072	7,257	901
R-squared	0.149	0.181	0.201

Note

*, ** and *** are significance levels of 10%, 5% and 1% respectively, with robust standard errors in brackets.

In fact, such a conclusion is in line with common perceptions. When corporates enter the mature stage, the growth of their market share begins to slow down, and sustainable development gradually becomes the corporate operation goal. In order to maintain reputation accumulation and corporate image, corporates often choose to respond more actively to the government call for sustainable development and improve their ESG performance level. This behavior can lead to more reputational resources and more revenue for the corporate, which in turn can feed into the quality development of corporates.

However, for the corporates in the growth stage, they are more inclined to prioritize business expansion and securing financial support, leaving them with fewer resources to follow the government sustainable development initiatives. On the other hand, when a corporate enters the decline phase, its operating capacity and profitability are relatively weaker, and risks such as lack of capital and shrinking market share start to emerge. The priority for corporates at this time is often to address the challenge of survival. At this point, improving ESG performance does not bring huge profits in the short term, nor does it improve the financing constraints. Therefore, the government sustainable procurement behavior on the impact of ESG performance is also weak.

#### 5.3.3 Differences in time window

The above regression examines the current impact of SPP implementation on corporate ESG performance, but the demonstration effect of government may have a long-term impact. We refer to Ma et al. (2016) [72] and Liu et al. (2022) [73] to calculate the moving average of the explanatory variables and the explanatory variables over 2, 3, 4 and 5 years, so as to explore the long-term impact of SPP on corporate ESG performance. The results are shown in Table 11, we can see that after the moving average treatment of the 2-year, 3-year and 4-year periods, there is still a continuous contribution of SPP behavior to the ESG performance over time. With the extension of the investigation period, the effect of government on corporates gradually weakened, and its impact faded with time until it gradually lost significance after 5 years. This suggests that the implementation of sustainable procurement by the government has a long-term impact on promoting ESG performance in corporates, however, due to the limitations of the sample time span, we were unable to examine the longer-term impact.

**Table 11:** Results of heterogeneity analysis based on time window.

	(1)	(2)	(3)	(4)
VARIABLES	Moving average 2 years	Moving average 3 years	Moving average 4 years	Moving average 5 years
spp	0.191***	0.126**	0.058*	0.011
	(0.057)	(0.062)	(0.029)	(0.115)
tobinq	-0.035***	-0.040***	-0.055***	-0.059***
	(0.006)	(0.006)	(0.011)	(0.010)
profit	1.595***	1.894***	1.601***	1.651***
	(0.202)	(0.223)	(0.213)	(0.294)
age	0.023***	0.024***	0.024***	0.025***
	(0.003)	(0.003)	(0.003)	(0.003)
ass_lia	0.221*	0.276**	0.248**	0.224*
	(0.116)	(0.116)	(0.109)	(0.114)
cash_ratio	0.496***	0.468***	0.536***	0.545***
	(0.143)	(0.159)	(0.154)	(0.192)
big_ratio	0.008***	0.008***	0.007***	0.007***
	(0.001)	(0.001)	(0.001)	(0.001)
Industry fixed effect	Yes	Yes	Yes	Yes
Year fixed effect	Yes	Yes	Yes	Yes
Constant	4.899***	4.955***	4.910***	5.054***
	(0.126)	(0.129)	(0.136)	(0.149)
Observations	15,101	11,703	9,261	5,975
R-squared	0.192	0.216	0.224	0.233

Note

*, ** and *** are significance levels of 10%, 5% and 1% respectively, with robust standard errors in brackets.

## 6. Discussion, conclusion, implication and future research

### 6.1 Discussion and conclusion

From the results of this paper, we find that an increase in the implementation of SPP can indeed enhance the corporate ESG performance in a region. Specifically, an increase of 1% of SPP implementation contributes to an increase of 0.18% corporate ESG performance. These findings add to the evidence that sustainable governmental behavior could influence the sustainable development of corporate [11]. Our results are also related to studies documenting how public procurement practices affect corporate behavior, such as corporate employment, profitability, and investment (e.g., [74–76]).

Second, we found that some corporate characteristics could also affect corporate ESG performance, which confirms that finding emphasized by extant literature. From the coefficient of control variables, we can see that the asset-to-liability ratio, the ratio of cash to total assets, and the ratio of net profit to total assets have a significant positive impact on ESG performance. In addition, with the increase of the corporate age, the corporate ESG performs better for those with longer operation period, and these results are consistent with the findings from the US [23] and German [77]. These findings will provide further insights into the factors that facilitate or impede resource dependency and its impact on the implementation of sustainable supply chain management initiatives [34, 78–79].

Last but not least, from the heterogeneity tests, we further explored the mechanisms of government leading effects behind different areas of China that might function differently. The eastern and central regions of China have a higher economic development and a better institutional environment [65–67]. These factors contribute to demonstrating SPP in the eastern and central regions and promote the sustainable development of corporates. Regional differentiation and inequality in the supply and development of public goods have long been heated topics in China [80, 81], and future research can further explore this topic as well as the mechanisms and reasons behind this phenomenon. Besides, mature corporations own more abundant resources and pay more attention to long-term sustainable development goals, so the sustainable behavior of public procurement will play a more active demonstrating role in improving corporate ESG performance. In addition, our analysis of the heterogeneity of the time window shows that the implementation of sustainable procurement by the government will promote corporate ESG performance in the long run, which also provides more evidence for the study of the temporal influence of government behavior [82].

### 6.2 Theoretical and managerial implications

The conclusions of this paper provide some theoretical and managerial implications.

First, this study centers on the relationship between SPP and corporate ESG performance, which responds to the call of Gantchev et al. (2022) [15] for research the leading role of government procurement in promoting corporate sustainable business practices. The findings presented above enable us to gain a deeper understanding of how the sustainable corporate behavior is affected by government actions such as public procurement.

Second, considering RDT has rarely been applied in a public procurement context [83], we quantitatively analyzed the impact of the public procurement on corporates, responding to Wontner et al. (2020)’s [16] suggestion for further research into the RDT in different and wide contexts.

By taking China as the research background, this study also responds to the call of Geng and Doberstein (2008) [84] and Walker et al. (2012) [85] for research in developing countries. We found that, despite of the differences in the political system and cultural background between developing and developed countries, government behavior plays an important role in corporate ESG performance [15]. These findings, based on the Chinese data, have enriched the related literature and will encourage more researches on sustainable development issues in developing countries.

From the managerial and policy implication aspects, as the implementation of SPP can increase corporate ESG performance, government should employ the “invisible hand” more effectively and participate in market economy activities more intelligently. Specifically, SPP policy should be added to government policy tool box to improve corporate ESG performance in addition to disclosure requirement, and the SPP policy employed should in particular attend to the “missing sectors” or “missing aspects” of sustainability in SPP (such as energy, transport etc.) identified in Grandia and Kruyen (2020) [86] and Cao et al.(2022) [52] for the good of corporate ESG performance; In addition, governments cannot simply administer a “one-size-fits-all” approach to promote corporate ESG performance, but rather, to adopt a differentiated policies tailored to the region’s economic development conditions and different stages of the corporate life cycle. Finally, it is important to strengthen the long-term assessment of the corporate sustainable behavior so that the government can play a more long-term demonstration and leading role.

### 6.3 Limitations and future research

Although the design and research of this study were conducted systematically and rigorously, our study still has its limitations that provide directions and ideas for future research.

First, the current research on the demonstration effect of the SPP on ESG performance is confined to one certain region, and future research could focus on the “spillover effect” of the region on the surrounding areas [87]. Second, the SPP data utilized for this study is based on conventional procurement, future study could use SPP data of public-private partnerships (PPP) projects. The potential effect of SPP of PPP projects on corporate ESG performance may be more pressing for study given the magnitude of PPP projects and their social, environmental, economic and political significance in an economy. Last but not least, relevant studies could also be conducted in different countries, especially between developing and developed countries, given that they have assumed common but differentiated emphasis on the ESG evaluation system [88].

## Supporting information

S1 Dataset (XLS)
